# Pectoralis Minor Syndrome: Subclavicular Brachial Plexus Compression

**DOI:** 10.3390/diagnostics7030046

**Published:** 2017-07-28

**Authors:** Richard J. Sanders, Stephen J. Annest

**Affiliations:** The Department of Surgery, University of Colorado Health Science Center, Aurora, Colorado and Presbyterian-St. Lukes Hospital, Denver, CO 80202, USA; stephenannest@comcast.net

**Keywords:** neurogenic thoracic outlet syndrome, NTOS, thoracic outlet syndrome, TOS, pectoralis minor syndrome, PMS, neurogenic pectoralis minor syndrome, NPMS, numbness and tingling, pain in neck and arm, occipital headache

## Abstract

The diagnosis of brachial plexus compression—either neurogenic thoracic outlet syndrome (NTOS) or neurogenic pectoralis minor syndrome (NPMS)—is based on old fashioned history and physical examination. Tests, such as scalene muscle and pectoralis minor muscle blocks are employed to confirm a diagnosis suspected on clinical findings. Electrodiagnostic studies can confirm a diagnosis of nerve compression, but cannot establish it. This is not a diagnosis of exclusion; the differential and associated diagnoses of upper extremity pain are always considered. Also discussed is conservative and surgical treatment options.

## 1. Introduction

Brachial plexus compression occurs either above the clavicle in the thoracic outlet area or below the clavicle under the pectoralis minor muscle (PMM). Because the symptoms of the two conditions are similar, the history and physical examination is the same for neurogenic thoracic outlet syndrome (NTOS) and neurogenic pectoralis minor syndrome (NPMS). The combination of paresthesia in the hand and pain in the arm should raise the question of brachial plexus involvement. Detailed history and physical examination are needed to determine whether brachial plexus compression is above the clavicle in the thoracic outlet area or below the clavicle, beneath the pectoralis minor muscle. In many patients, the two conditions coexist. No diagnostic test is pathognomonic for NTOS or for NPMS. Evaluation should begin with the clinical picture [1].

## 2. Anatomy

**Figure 1:**
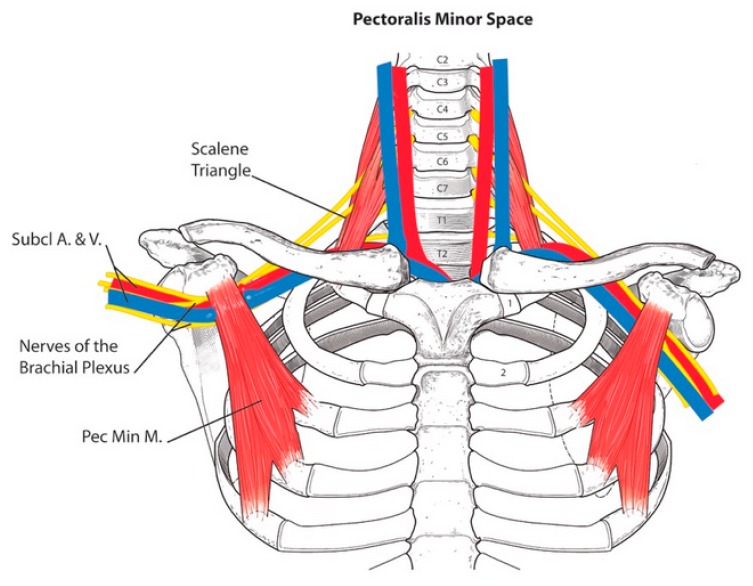
The anatomy of thoracic outlet and pectoralis minor areas. The scalene triangle is above the clavicle. Between the anterior and middle scalene muscles are the five nerve roots and trunks of the brachial plexus and the subclavian artery. The subclavian vein runs anterior to the triangle. Below the clavicle the axillary artery and vein lie immediately under the pectoralis minor muscle. The cords and branches of the brachial plexus usually surround the axillary artery. Figure 1 is reprinted with permission from Sanders R.J. and Haug C.E.: Thoracic outlet syndrome: A common sequela of neck injuries; Lippincott: Philadelphia, PA, USA [2]. Abreviations: Subcl A.&V., subclavian artery and vein; Pec Min M., pectoralis minor muscle.

The anatomy of the structures around the brachial plexus, both above and below the clavicle, are seen in Figure 1. The scalene triangle lies in the thoracic outlet area; the pectoralis minor muscle lies immediately below the clavicle, above the brachial plexus and axillary vessels.

**Figure 2:**
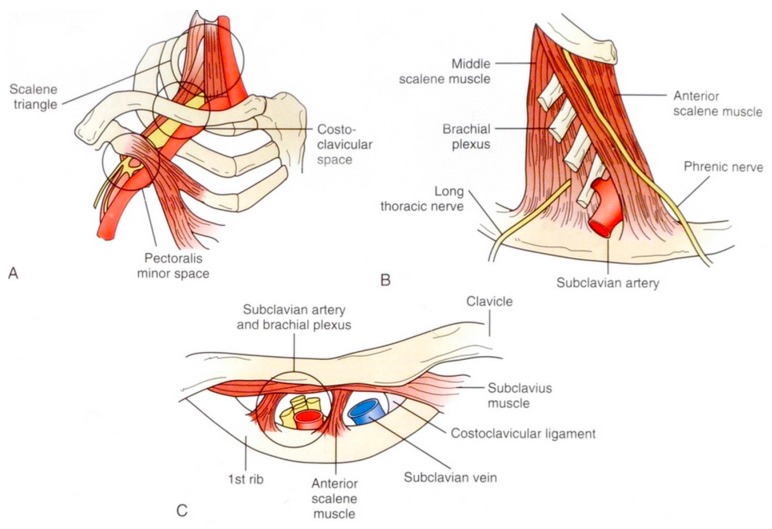
The three anatomical spaces for the neurovascular bundle. (**A**) Pectoralis minor space; (**B**) Scalene triangle; (**C**) costoclavicular space. Reprinted with permission from Sanders R.J. and Haug C.E.: Thoracic outlet syndrome: A common sequela of neck injuries; Lippincott: Philadelphia, PA, USA [2].

Three anatomical spaces can be identified through which the neurovascular bundle passes. The bundle consists of the triad of brachial plexus, subclavian artery, and subclavian vein. The bundle passes from above the clavicle in the scalene triangle, directly under the clavicle in the costoclavicular space, and below the clavicle under the pectoralis minor muscle (Figure 2 [2]).

## 3. History

History should begin with a list of current symptoms. Here, current refers to symptoms that have been present for the past few weeks. Once current symptoms have been established, the onset of these symptoms is discussed.

The onset starts with the very first symptoms and what was happening when they occurred. Was there some type of accident, repetitive stress, or did it begin spontaneously? The purpose of this is to determine whether the etiology was a stretch injury of the scalene or pectoralis minor muscles resulting in muscle fibrosis and brachial plexus nerve entrapment; or could there have been a direct nerve stretch injury.

### 3.1. Trauma

Many patients have a history of a traumatic incident, such as a motor vehicle accident or a fall down stairs, on ice or a slippery floor [2]. In the absence of history of an injury, patients should be asked about their occupation, exercise habits, and sports participation, looking for a cause of repetitive stress injury (RSI). RSI occurs in many forms. Working on assembly lines or keyboards are well recognized causes of RSI.

Sports involving a throwing or lifting motion can also produce RSI. However, this RSI causes not only NTOS but is a major cause of NPMS. Because the pectoralis minor muscle attaches to the coracoid process of the scapula, repetitive arm and shoulder movements are the usual etiology of NPMS. This is particularly true in teenagers and young adults who participate in competitive sports. Sports that have been seen to cause NPMS include swimming, baseball (especially pitchers), volleyball, weightlifting, and other activities that have in common scapular retraction stretching the pectoralis minor muscle (PMM) [3].

The history should include whether or not, prior to the onset of the present illness, previous accidents or similar symptoms had occurred. This includes previous trauma or previous surgery for similar symptoms.

### 3.2. Nerve Injury

The time symptoms develop following an accident is important to document. Paresthesia and/or weakness that occurs immediately after an injury can be due to spinal cord shock or to stretch injuries of nerves of the brachial plexus. If the nerve symptoms disappear in the first few days, spinal shock was the most likely diagnosis. If the symptoms persist, direct nerve injury is likely. In contrast to nerve injury, torn muscles producing nerve compression are the usual cause of paresthesia that develops a few days to many months after the accident and usually progresses over time.

### 3.3. Spontaneous

A minority of patients have no history of trauma or a specific incident that heralded the onset of symptoms. In such patients, a cervical rib or anomalous first rib can be the etiology. While the majority of patients with rib abnormalities remain asymptomatic throughout their lives, a few of such patients will develop symptoms spontaneously. This is easily detected with a plain cervical spine or chest X-ray.

Once the order of onset of symptoms has been established, the progression or regression of symptoms is determined. This may be related to the treatment provided to date. When recommending therapy to a patient, it is important to know previous treatment and its results. If a patient has already received physical therapy (PT), the specific modalities should be included as some modalities of PT are more helpful than others for NTOS and NPMS.

## 4. Symptoms

Pain, numbness and/or tingling, and weakness are the common symptoms.

### 4.1. Pain

The locations of pain are more helpful in the diagnosis than a patient’s description of the quality of the pain. Whether the pain is described as “muscular” or “nerve” pain may occasionally help, but, seldom matters. Neck pain and occipital headaches are common in NTOS. Pain or tenderness in the axilla and the anterior chest wall just below the clavicle, strongly suggest NPMS. Pain in the shoulder, upper arm, and forearm is frequent in both of these conditions.

### 4.2. Paresthesia

Paresthesia is present in over 90% of NTOS and NPMS patients. It usually involves all five fingers, but commonly is worse in the ulnar nerve distribution involving the 4th and 5th fingers.

### 4.3. Weakness

Weakness is frequently seen, but not as often as pain and paresthesia. Signs of weakness are dropping things and poor grip.

The symptoms in patients with NPMS Alone compared to patients with combined NPMS and NTOS are presented in Table 1. NPMS Alone patients had significantly fewer occipital headaches and pain in the neck, supraclavicular area, and shoulder. When they did have occipital headaches and neck pain, they were usually mild. This was the main difference between the two groups. Weakness occurred less often in the NPMS Alone patients. Significantly more NTOS Alone patients were still working (Table 1) [3].

## 5. Physical Examination

### 5.1. Tenderness and Tinel’s Sign

Physical examination includes investigation for brachial plexus compression at the scalene triangle and under the pectoralis minor muscle. Additionally, evidence is sought for nerve compression at the elbow, forearm, and wrist. These signs include both tenderness and Tinel’s sign in each location (Table 2 and Table 3).

### 5.2. Provocative Maneuvers

In addition to palpating for tenderness and checking for positive Tinel’s and Phelan’s sign, four maneuvers are specific for identifying brachial plexus compression. These are labeled provocative maneuvers and include [4].

#### 5.2.1. Neck Rotation

**Figure 3:**
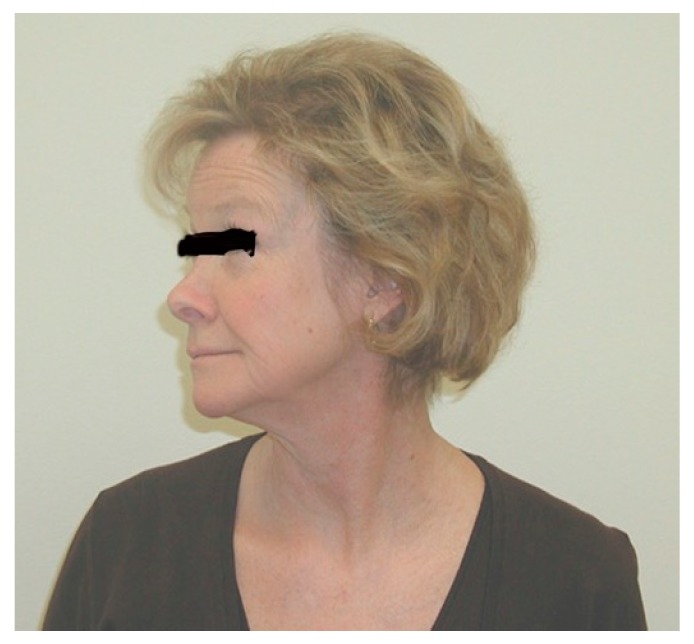
Neck rotation, chin toward shoulder.

This is performed by rotating the chin as far as possible towards one shoulder. (Figure 3) Normally, this elicits no symptoms. In NTOS patients, this causes symptoms of pain and/or paresthesia on the opposite (contralateral) side. Symptoms elicited on the same (ipsilateral) side suggest cervical spine disease. This is performed in each direction (to the right, then the left).

#### 5.2.2. Head Tilt

**Figure 4:**
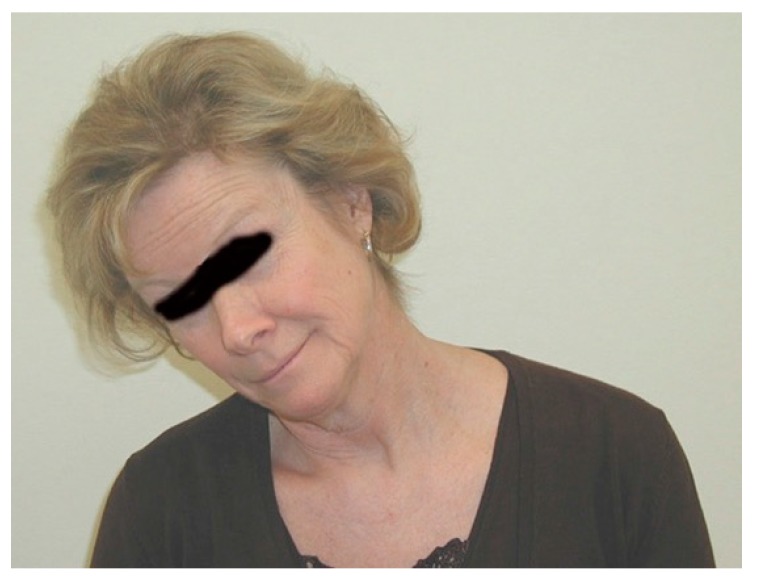
Head tilt, ear to shoulder.

Tilting the head by dropping the ear toward the shoulder normally elicits no symptoms. In NTOS patients, this causes symptoms of pain and paresthesia in the opposite extremity. Symptoms elicited in the same extremity suggest cervical spine disease. Patients with NPMS usually have minimal or no response to neck rotation and head tilt. This maneuver is performed in each direction (Figure 4).

#### 5.2.3. Upper Limb Tension Test (ULTT)

**Figure 5:**
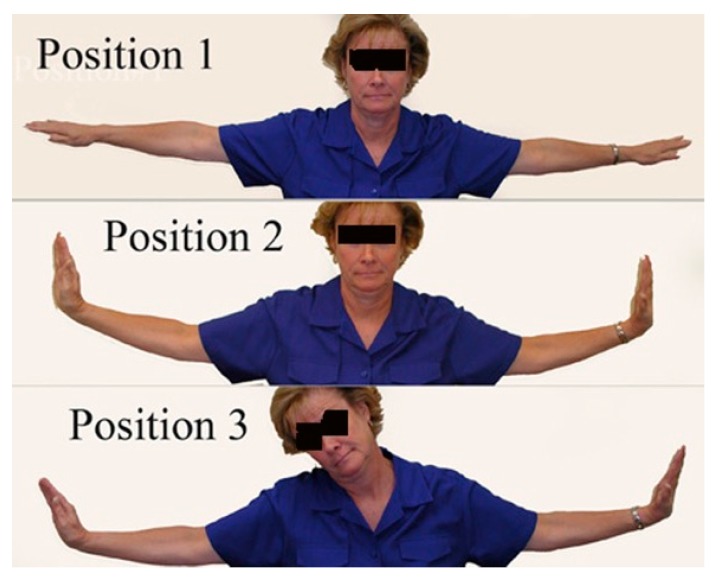
Upper Limb Tension Test (ULTT).

This test is performed by executing three steps: First, the elbows are extended and the arms elevated to 90° parallel to the floor (Figure 5). The second position is dorsiflexion of the wrists. The third position is tilting the head, ear to shoulder, first to the right and then to the left. Normally, no symptoms are elicited. When there is brachial plexus compression, this maneuver brings on the patient’s symptoms of pain and paresthesia within a few seconds. The earlier the response, the stronger is the degree of compression; that is, a positive response in position 1 means a stronger degree of compression than just a positive response in position 3 with no responses in positions 1 and 2. This test is comparable to straight leg raising in the lower extremity. It was first described by Elvey and has been modified [5].

#### 5.2.4. 90° ABD or EAST (Elevated Arm Stress Test)

**Figure 6:**
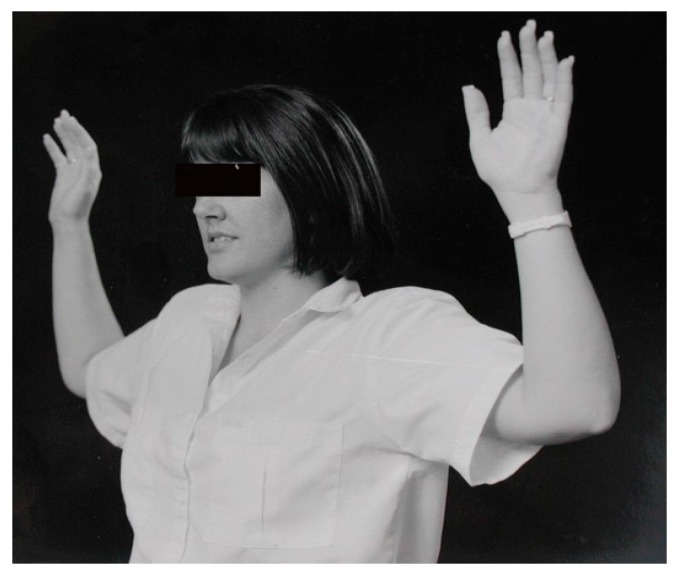
90° Abduction in External Rotation (90° AER).

Abducting the arms to 90° in external rotation (Figure 6). This is also referred to as the Elevated Arm Stress Test or the Roos test. Opening and closing fingers is not necessary. It is the elevation and compression of the neurovascular bundle that is being tested. Finger exercise is indicated only for arterial TOS, when checking for claudication. A positive response is the onset of pain or paresthesia within 60 s. In moderate to severe compression, symptoms often appear within 5 to 30 s.

## 6. Numbness

Physical examination includes checking for reduced sensation to light touch in the fingers, comparing one hand to the other. While this can be done with cotton, it can also be done by using the index fingers of the examiners hands lightly touching the bilateral finger tips of the patients hands simultaneously. Each of the five fingers is tested separately. A positive finding is demonstrating reduced sensation to light touch in one or more fingers of one hand compared to the other. The finding of decreased sensation may be present in only some fingers, such as the fourth and fifth fingers.

Findings on physical examination in NPMS Alone patients compared to combined NPMS and NTOS patients are presented in Table 4. While there was no significant difference between NPMS Alone and combined NPMS and NTOS patients in pectoralis minor tenderness, NPMS Alone patients had significantly fewer positive physical findings in all other areas (Table 4) [3].

## 7. Diagnostic Tests

### 7.1. Muscle Blocks

Muscle blocks must be distinguished from brachial plexus blocks. A plexus block makes the arm pain free, but also renders it numb and weak. The muscle block relaxes the muscle so it no longer compresses the nerves. After each the muscle block, the physical examination, including the provocative maneuvers, is repeated and the degree of reduction in pain, tenderness, numbness, tingling and weakness. Is recorded.

### 7.2. Scalene and Pectoralis Minor Muscle Blocks

Injection of 1% Lidocaine into the PMM and ASM is a very useful test to confirm a diagnosis of NTOS and NPMS. These two conditions can exist alone or together. Since becoming aware of NPMS, it has been found that coexistence occurs in at least 75% of the patients seen for NTOS. Therefore, it is important during the physical examination to check for tenderness in both the anterior scalene muscle (ASM) and pectoralis minor muscle (PMM) areas.

The indication to perform a muscle block is a history of pain and elicitation of tenderness over the ASM and/or PMM. When there are positive symptoms and signs in both areas, a block of each muscle is performed during the same examination. The blocks are performed separately; the pectoralis minor block being done first, followed by a repeat physical examination. If all symptoms and signs are gone, the scalene block is not performed. If some symptoms or signs persist, a scalene block is performed and the physical exam repeated again.

### 7.3. Technique of Muscle Block

The block can be performed with the patient recumbent or sitting. Ultrasound can be used to locate the muscle. In the absence of ultrasound, and with experience, the block can also be performed successfully using standard landmarks. The pectoralis minor landmark is 4–6 cm below the clavicle and over the most tender spot of the PMM; for the scalene, the landmark is through the most tender spot 1–3 cm lateral to the side of trachea and 2 cm above the clavicle. The entry point is always lateral to the carotid pulse and usually through the clavicular head of the sternocleidomastoid muscle. One percent Lidocaine is used for diagnostic blocks because it is short acting. The effect is usually noted within 60 s and usually lasts about 30 min. Should side effects occur from the block, they are gone in a short time.

Pectoralis minor muscle block is performed by injecting 4 mL of 1% Lidocaine through a 5 mL syringe with a 1.5 inch needle. The needle is directed upwards, at a 45° angle, to avoid entering the pleura. The needle entrance is located at the most tender spot 4–5 cm below the clavicle in the region of the PMM. The Lidocaine is injected over an area 1–2 cm deep, and 2 cm wide, by injecting 0.3 to 0.5 mL at a time and moving the needle after each small injection to cover the area. The syringe is aspirated each time the needle is moved to prevent injecting into a blood vessel. If blood is aspirated, the needle is withdrawn a few mm and repositioned in a slightly different direction to avoid the blood vessel. A successful block is indicated by loss of tenderness in the area. If tenderness persists, the procedure can be repeated, aiming the needle in a slightly different direction. Following a successful block, the physical examination is repeated and results recorded. Side effects of the block include increased paresthesia and weakness from Lidocaine spreading to portions of the plexus. These usually wear off in 5–10 min.

The scalene muscle block is performed in similar fashion. Again, aiming the needle cephalad at a 45 degree angle is done to avoid a pneumothorax [1]. Important: where the patient’s clavicle projects to make it difficult to achieve a 45 degree angle, the needle is bent to keep the direction cephalad enough to avoid a high lying pleura. When a good block is confirmed by loss of tenderness in the area, the physical examination is repeated once again. Side effects of the scalene block include temporary hoarseness from Lidocaine spreading to a laryngeal nerve or Horner’s syndrome due to spread to the sympathetic chain. The authors have not experienced diaphragmatic paralysis.

## 8. Electrodiagnostic Studies

### 8.1. Electromyography (EMG)

Until the 1990s, EMG studies were described as normal in most NTOS and NPMS patients. In 1993, measurement of the medial antebrachial sensory cutaneous nerve was introduced [6,7]. Over the next 15 years, further refinement of this technique and normal ranges were developed. This test has proved positive in the large majority of patients operated upon for NTOS and NPMS [8,9].

### 8.2. C8 Nerve Root Stimulation

This test, though very helpful, is seldom used because it is more painful than most other nerve measurements. It is performed by direct stimulation of the C8 nerve root just outside the cervical spine in the posterior neck. It measures conduction time between the C8 nerve root and a point selected in the neck or arm [9,10]. 

### 8.3. Other Tests

#### 8.3.1. A Few Other Techniques Have Been Tried

These have been helpful in occasional cases but not in the majority of NTOS or NPMS patients. These include MRI of the brachial plexus [11] and neurography [12].

#### 8.3.2. X-rays

A plain X-ray of the neck or chest should always be performed to determine whether there is a cervical rib or anomalous first rib. An apical lung mass is rarely discovered (Pancoast tumor).

#### 8.3.3. Associated and Differential Diagnosis

Several conditions can exist with brachial plexus compression as associated conditions and must also be differentiated from it. These conditions are listed in Table 5.

## 9. Double Crush Syndrome

It is quite common for a second or even a third area of compression to accompany brachial plexus compression. This has been termed “double crush syndrome” [13] or “Triple Crush”.

## 10. Separating NTOS from Neurogenic Pectoralis Minor Syndrome (NPMS)

NTOS usually includes symptoms of neck pain and occipital headaches. NPMS usually includes symptoms of chest pain below the clavicle and in the axilla. Physical examination for NTOS includes significant tenderness over the scalene muscles and radiating pain with pressure over the brachial plexus above the clavicle, and Tinel’s sign over the brachial plexus in the neck. NPMS usually has tenderness over the subclavicular area in the region of the PMM and in the axilla. When physical examination includes positive findings in both areas the two conditions usually co-exist. It should be noted that about 75% of the patients seen for NTOS also have symptoms and physical findings of NPMS.

## 11. Clinical Diagnosis of NPMS Alone Verses NPMS with NTOS

Patients with NPMS Alone usually have a history of repetitive stress activities, particularly competitive sports involving the upper extremities. These include swimming, throwing, volley ball, and weight lifting. Patients with both NTOS and NPMS have histories of motor vehicle accidents or falls down stairs or on ice. In the combined patients, tenderness is usually worse over the anterior chest wall, axilla, and trapezius with less tenderness in the supraclavicular area. 

Patients with NTOS alone are more likely to have no headache or neck pain. If these are present, they are minor symptoms.

Physical examination in NPMS Alone patients usually includes tenderness over the pectoralis minor muscle and axilla with little or no tenderness over the scalenes.

## 12. Treatment of NTOS

**Figure 7:**
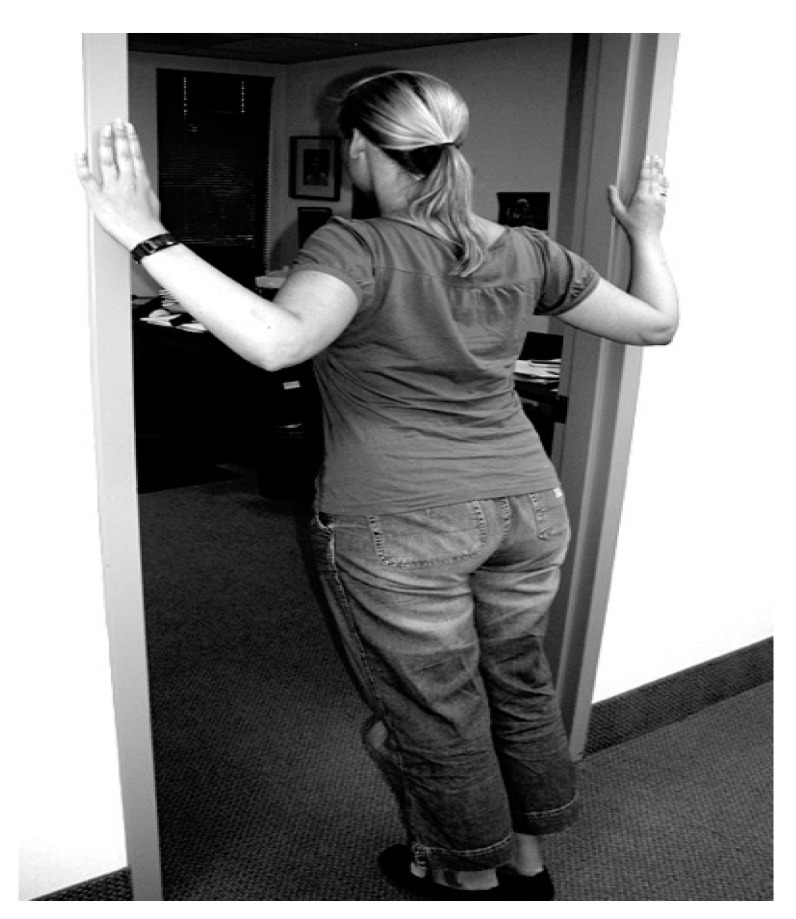
Pectoralis minor stretching. Standing in an open doorway with hands on door jams at shoulder level. Letting the body fall forward stretches the pectoralis minor muscles. The degree of stretching is controlled by the position of the feet either closer to or farther from the threshold line.

Conservative Treatment. Pectoral minor muscle stretching is the most important technique for treating NPMS. This should be recommended for all patients diagnosed with NPMS. It can be performed in a few different ways. We have found standing in an open doorway, with the hands at shoulder level resting on the door jams, is an effective way of achieving this (Figure 7). Patients are instructed to stretch three times a day, hold each stretch for 15 to 20 s, rest for the same length of time, and do three repeats at each session. We suggest three sessions a day, seven days a week. This should be performed for three months. If patients improve enough to be comfortable, nothing else need be done. If there is no significant improvement in three months, they can be offered a pectoralis minor tenotomy, a simple, minimal risk outpatient procedure.

## 13. Surgical Treatment

Currently, results of pectoralis minor stretching are antidotal. A number of patients in whom NPMS was their only diagnosis have achieved good improvement with just one or two months of stretching. These patients continue to do well. The patients in this category had symptoms for less than a year. As part of their treatment, patients stopped the repetitive activity that had elicited their symptoms. Later, they were able to return to that activity, but with less intensity. 

Surgical treatment is pectoralis minor tenotomy with partial myomectomy. This can be achieved through either a transaxillary or thoracic approach. Our preference is for the transaxillary approach as it permits wider exposure to completely excise the clavipectoral fascia and any bands or accessory muscles, such as Langer’s axillary arch [14], around the axillary neurovascular bundle.

Technique through the axilla begins with a 4–7 cm transverse incision 1–2 cm above the bottom of the axillary hairline, beginning at the anterior axillary fold. The pectoralis major muscle is retracted and the pectoralis minor identified by its attachment to the coracoid process. The origin of pectoralis minor is divided at the coracoid, a 1–2 cm section of the detached muscle end is excised, to prevent reattachment to the brachial plexus. Care is taken to preserve the lateral pectoral nerves to pectoralis major. These nerves traverse through the pectoralis minor and dividing them leads to atrophy of the pectoralis major. Before closure, the clavipectoral fascia and any bands or muscle fibers are excised to leave the neurovascular bundle free of any compressing tissue.

Postoperatively, the remaining body of the pectoralis muscle adheres to the anterior chest wall. To facilitate this adherence, the patient should avoid elevating the arm above shoulder level for 2–3 months after surgery. However, once a day the patient should elevate the arm 180° to avoid a frozen shoulder.

## 14. Results of Surgical Treatment

Results for pectoralis minor release depend upon whether or not it is associated with NTOS. When NPMS is the only diagnosis, results have been 85% successful. However, when NPMS and NTOS coexist, the success rate for pectoralis minor release alone is closer to 35%. The other 65% may need thoracic outlet decompression at a later date [1,3,15].

## 15. Oral Informed Consent

Figure 3, Figure 4, Figure 5 and Figure 6 were of employees of the senior author. Oral informed consent was provided to the author by each subject provided their eyes would be blacked out so identity would be protected.

## Figures and Tables

**Table 1:** Symptoms of pectoralis minor (PM) alone and with neurogenic thoracic outlet syndrome (NTOS).

	PM Alone	PM + TOS	*p* Value
Number patients	39	37	
Number operations	52 (*N* = 52)	48 (*N* = 52)	
Pain	(No)	(No)	
Occipital Headache	31% (16)	81%(39)	<0.001 *
Neck	50% (26)	96% (46)	<0.001 *
Supraclavicular area	44% (23)	79% (38)	0.004 *
Trapezius	87% (45)	96% (46)	0.163
Pec Minor (Ant. Chest)	69% (36)	92% (44)	0.059
Axilla	52% (27/50)	78% (29/37)	0.024 *
Shoulder	69% (36)	90% (43)	<0.001 *
Arm	71% (37)	88% (42)	0.053
Weakness	58% (30)	88% (42)	0.002 *
Paresthesia	88% (46)	98% (47)	0.114
All 5 fingers	54% (28)	48% (23)	0.699
4th and 5th	29% (15)	42% (20)	0.67
1st–3rd	6% (3)	8% (4)	0.71
None	12% (6)	2% (1)	0.11
Still working	85% (33/39)	57% (21/37)	0.011 *

* ≤0.05, statistically significant difference.

**Table 2:** Areas of tenderness.

Signs of Nerve Compression Are the Following:
Tenderness over:
1. Anterior scalene muscle (ASM) and brachial plexus (BP) just above the clavicle
2. Pectoralis minor muscle (PMM) located about one inch below the lateral portion of the clavicle
3. Axilla (associated with pectoralis minor compression)
4. Medial epicondyle over the ulnar nerve (cuboid tunnel)
5. Pronator tunnel
6. Radial tunnel
7. Carpal tunnel
8. Cervical and thoracic spine
Signs of inflammation are tenderness over:
1. Biceps and rotator cuff tendons
2. Trapezius and rhomboid muscles

**Table 3:** Tinel’s sign.

Tinel’s Sign is Tested in the Following Areas:
1. Supraclavicular area over the brachial plexus
2. Elbow over the ulnar nerve
3. Pronator tunnel (median nerve)
4. Radial Tunnel (radial nerve)
5. Carpal Tunnel, where Phelan’s sign is also tested
6. Guyen’s canal (ulnar nerve at wrist)

**Table 4:** Physical exam–incidence of positive responses.

	PM Alone	PM + TOS	*p* Value
Number patients	39	37	
Number operations	52 (*N* = 52)	48 (*N* = 52)	
Pain	(No)	(No)	
Pec Minor tenderness	92% (48)	100% (48)	0.119
Trapezius tenderness	56% (29)	88% (42)	
Axilla tenderness	71% (32/44)	95% (38/40)	0.008
90° AER	82% (40/50)	100% (45/46)	0.008
ULTT of Elvey	79% (40)	92% (46)	0.008
Scalene tenderness	48% (24)	86% (43)	<0.001
Biceps tenderness	54% (28)	88% (44)	<0.001
Neck rotation	40% (22/51)	80% (41)	<0.001
Head tilt	49% (24/51)	76% (39)	<0.001
Decr. Sensation to Touch	31% (16/51)	48% (23)	<0.001

**Table 5:** Differential and associated diagnoses.

Cervical Spine Disease
Cervical spine strain
Shoulder pathology
Fractured clavicle
Cuboid Tunnel Syndrome (Ulnar nerve entrapment at elbow)
Pronator Tunnel Syndrome
Radial Tunnel Syndrome
Cervical spine disease
Parsnips-Turner Syndrome
Stretch injury of the brachial plexus
Pancoast Tumor
